# Quantifying the Impact of Headlamp Light Distribution on Automotive Camera Perception: Establishing a New Primary Design Parameter

**DOI:** 10.3390/s26113290

**Published:** 2026-05-22

**Authors:** David Hoffmann, Julian Lerch, Korbinian Kunst, Nikolai Kreß, Tran Quoc Khanh

**Affiliations:** Laboratory of Adaptive Lighting Systems and Visual Processing, Technische Universität Darmstadt, Hochschulstr. 4a, 64289 Darmstadt, Germany; lerch@lichttechnik.tu-darmstadt.de (J.L.); kunst@lichttechnik.tu-darmstadt.de (K.K.); kress@lichttechnik.tu-darmstadt.de (N.K.); khanh@lichttechnik.tu-darmstadt.de (T.Q.K.)

**Keywords:** automotive lighting, headlamp evaluation, camera signal-to-noise ratio, perception benchmark, region of interest, driver assistance systems, machine vision

## Abstract

Perception-oriented evaluation of automotive headlamps still relies mainly on human-vision photometric criteria, although forward-facing cameras are increasingly safety-critical sensing elements for night driving. This paper benchmarks 16 measured production headlamp light distributions with a simulation chain that combines headlamp spectra and beam patterns, diffuse scene reflection, an imaging-transfer model, and an EMVA-based camera model. The quantitative chain maps scene radiance to sensor-domain signal-to-noise ratio, derives task-specific required signal-to-noise curves from a six-network object-recognition ensemble, and aggregates local threshold satisfaction as region-of-interest coverage across three target reflectances and five driving speeds using WLTP moving-time weights. For the baseline RGB camera, WLTP-weighted coverage ranges from 18.95% to 53.48% across the evaluated light distributions, corresponding to a factor of 2.82 between the weakest and strongest distribution. The camera-parameter sweeps show that favorable beam placement can deliver comparable benchmark coverage with roughly 60% smaller pixel pitch than the weakest distribution, corresponding to an 84% reduction in pixel area, or at materially shorter exposure times. The WLTP-weighted coverage score correlates positively with the established Headlamp Safety Performance Rating, with Pearson r=0.68 for the RGB configuration, indicating partial alignment between human-centric and camera-centric illumination needs while confirming that the metrics are not interchangeable. The results identify headlamp light distribution as a primary design parameter for nighttime camera perception and provide a quantitative basis for co-design of automotive lighting and camera-based systems.

## 1. Introduction

Forward-facing cameras are now a primary sensing modality for advanced driver-assistance systems and automated-driving stacks, and their market relevance continues to grow with the expansion of camera-rich driver-assistance platforms and higher levels of driving automation [1,2]. Nighttime perception is therefore governed less by ambient light than by the irradiance available at the sensor from the vehicle’s own headlamps. Standard headlamp evaluation relies on human-vision photometric ratings such as the Headlamp Safety Performance Rating (HSPR), a standardized, laboratory-based assessment using human-centered weighting [3]. HSPR builds on the CIE performance-assessment method for vehicle headlighting systems [4], but neither framework addresses the signal quality required by camera-based perception. Headlamp evaluation metrics therefore quantify human-visibility outcomes, and no dedicated measure for camera signal quality in safety-critical scene regions exists.

This discrepancy is addressed through a simulation benchmark built from the components that isolate the dominant design dependencies. The benchmark starts from headlamp light distribution and source spectra, models scene objects as diffuse reflectors, maps the reflected light through an imaging model, predicts sensor-domain signal-to-noise ratio with an EMVA-based camera model, and evaluates whether safety-relevant scene regions satisfy task-derived signal requirements. The resulting metric, coverage, measures the fraction of the region of interest in which the required signal-to-noise ratio is achieved.

This work provides a camera-centered benchmark framework for headlamp-camera systems, quantifies the coverage spread across 16 measured production automotive light distributions, and quantifies relationships between beam pattern and key camera parameters for joint lighting-sensing design. The benchmark derives spatially varying signal-to-noise requirements from detection performance, replacing the common fixed-threshold approach with a continuously graded demand map across safety-relevant scene regions.

An extended thesis-level presentation of the benchmark framework and its broader research context is available in the corresponding author’s doctoral dissertation [5].

For nighttime machine perception, the headlamp beam pattern is not an environmental boundary condition but a first-order design parameter. Better light placement directly increases the photonic input available to the camera in safety-critical regions and can be traded against sensor-side requirements such as pixel size and exposure time. The resulting benchmark makes lighting quality a directly comparable engineering variable across existing systems and provides the quantitative basis for lighting-sensing co-design.

## 2. Related Work

### 2.1. Adaptive Lighting for Camera-Oriented Illumination

Automotive lighting is evolving from static low-beam and high-beam systems toward electronically controlled adaptive beam patterns. Adaptive Driving Beam systems, pixel-LED headlamps, and micro-LED concepts provide the spatial degrees of freedom needed to redistribute light toward task-relevant areas instead of only increasing total luminous flux [6,7,8]. This trend is directly relevant for driver assistance and automated driving because the illumination pattern presented to the camera can now be shaped as an active design variable. Perception-oriented beam concepts proposed by Kobbert and Erkan follow this direction by optimizing light placement with respect to object occurrence, gaze behavior, and visibility-oriented target zones rather than only by maximizing nominal roadway brightness [9,10].

Prior work indicates that adaptive illumination should be assessed not only in terms of human visibility and glare, but also in terms of how effectively it supports scene understanding. Matrix headlamp concepts demonstrate that concentrating light in relevant regions can preserve useful visibility at lower optical power [11], and perception-aware lighting approaches extend this principle toward closed-loop illumination using scene analysis and camera feedback [12,13]. Outside automotive lighting, automatic illumination planning for robotic inspection further shows that active light control can be optimized directly against image-quality objectives [14]. To the authors’ knowledge, no public comparison has yet provided a direct quantitative measure of how different beam patterns translate into camera-relevant performance metrics.

### 2.2. Nighttime Perception Robustness and the Co-Design Gap

On the perception side, modern vision models have improved substantially, from residual convolutional networks to efficient mobile backbones and transformer-style architectures [15,16,17,18,19,20]. At the same time, nighttime detection remains constrained by the quality of the captured signal. Prior work shows that detection performance degrades rapidly once the image enters a low-signal regime, especially for small or weakly contrasted objects [21,22,23,24]. Across that literature, reliable detection is commonly discussed in terms of broad fixed thresholds or ranges, typically around a linear signal-to-noise ratio of roughly 5 to 10, which is useful as a rule of thumb but too coarse for safety-differentiated benchmarking.

Camera designers optimize sensor-side parameters such as pixel size, gain, and read noise, whereas lighting developers optimize beam compliance, visibility, and glare. For nighttime machine perception, however, both sides act on the same signal path. A benchmark that explicitly links beam pattern, sensor-domain signal-to-noise ratio, and safety-relevant scene coverage is the prerequisite for lighting-sensing co-design. The present benchmark converts broad literature SNR thresholds into task-defined, spatially varying requirements derived from detection performance itself.

## 3. Materials and Methods

The benchmark follows a five-stage signal path from headlamp output to perception-relevant scene coverage. The evaluated light distribution and source spectrum illuminate diffuse target materials as objects on the road surface and pedestrian clothing, an imaging model maps the reflected radiance to the camera, an EMVA-based sensor model predicts signal-to-noise ratio, and the resulting signal quality is compared with task-derived requirements inside a safety-derived region of interest. This structure isolates the dominant design dependencies for comparing headlamp systems without claiming to be an exhaustive physical scene simulator.

### 3.1. Benchmark Architecture

**Figure 1:**
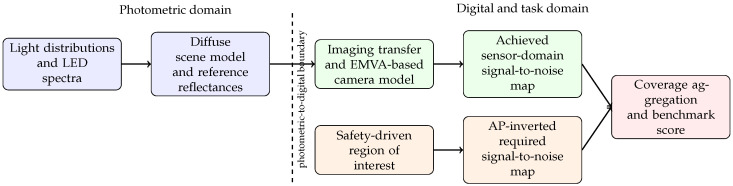
Signal-path view of the coverage benchmark. The figure traces the signal path from headlamp input through diffuse scene reflection and camera conversion to safety-weighted coverage, and marks the photometric-to-digital boundary.

The pipeline links five components: a dataset of 16 automotive headlamp light distributions as photonic input, a diffuse-reflection scene model with measured reflectance spectra, a validated EMVA-based camera model, a safety-derived region of interest that encodes detection priority as a function of driving speed and reaction urgency, and a controlled object-recognition study that maps signal-to-noise ratio to required detection performance. Coverage is defined as the fraction of the relevant scene in which the achieved signal-to-noise ratio exceeds the locally required threshold. The signal path is summarized in Figure 1.

### 3.2. Photometric Scene Modeling

The photometric stage defines the optical input presented to the camera. It combines the 16 benchmark light distributions with a controlled diffuse-reflection scene abstraction so that beam-pattern geometry and material reflectance remain the dominant free variables in the comparison.

#### 3.2.1. Light-Distribution Dataset

Each light distribution in the dataset was measured with a goniophotometer and stored in IES photometric format, providing a full-sphere angular description of the luminous intensity distribution. The inverse-square law is applied point-by-point to convert the IES intensity data into an illuminance field for every ground-plane position in the traffic space ahead of the vehicle. Isolux contours are derived directly from this field by connecting all positions that receive the same illuminance level, and the 3-lux threshold serves as the reference line throughout the benchmark. The dataset therefore captures the complete spatial allocation of the photon budget that each system presents to the camera before any sensor parameter is varied.

**Figure 2:**
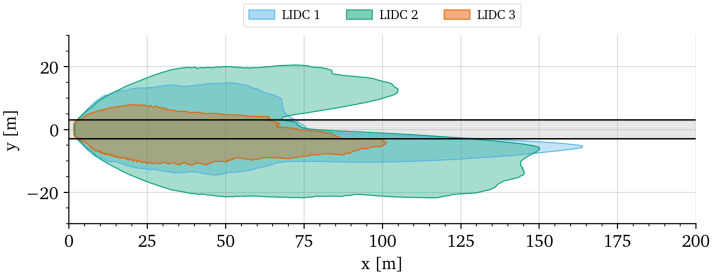
Representative 3-lux isolines of three headlamp light distributions. LIDC 1 is range-focused and projects a narrow elongated beam farther down the road. LIDC 2 is width-focused and distributes light broadly across the near and mid field. LIDC 3 represents a limited baseline with reduced longitudinal reach and lateral support. The figure grounds the benchmark in the beam-shape differences that define the photonic input to the automotive camera. The gray background depicts a two-lane road, each lane measuring three meters wide.

Figure 2 illustrates representative beam-shape archetypes used as benchmark inputs and shows how different headlamp systems allocate roadway illuminance before any camera parameter is varied. The 3-lux isolines provide an intuitive view of the photon budget presented to the perception system.

The evaluated set spans a broad quality range of current automotive headlamp systems. Each of the 16 light distributions was obtained from a production automotive headlamp system and is represented as a full-sphere goniophotometric measurement in IES format, which is also the format required for computing the Headlamp Safety Performance Rating. The systems are selected from available measured production distributions to span HSPR, reach, width, and beam-shape diversity, but they are not a statistically stratified market sample. Table 1 lists the 16 light-distribution identifier categories together with their headlamp safety performance ratings for low-beam, high-beam, and automatic operation. All distributions are evaluated with the same LED spectra so that the benchmark isolates differences caused by beam-pattern geometry and photometric allocation.

The HSPR scoring system was developed by the GTB Working Group on Special Vehicle Problems using the headlighting performance-assessment methodology of CIE technical committee TC4-45 [3,4]. It assigns each headlamp a numerical score based on a weighted evaluation of illuminance across standardized roadside measurement zones, capturing both longitudinal reach and lateral spread in a single scalar value. Six categorical rating levels are defined in ascending order: Standard, Good, Advanced, Excellent, Premium, and Premium+, so that a reader can interpret the table entries without consulting a separate reference. Low-beam and high-beam scores are computed independently, reflecting the distinct photometric requirements of each operating mode. The automatic-operation score combines low-beam operation with automatic high-beam activation when traffic conditions allow high beam, for example when no oncoming traffic is present. Higher scores in each column indicate systems that allocate photons more effectively across the relevant traffic zones, making the rating the primary human-centric reference for the correlation analysis in Section 4.

The raw IES files are proprietary and cannot be redistributed. The dataset composition is therefore characterized through non-reconstructive photometric descriptors for all 16 systems. These descriptors include iso-lux area, longitudinal reach, lateral width, angular peak intensity, angular spread, and field of view (FOV) estimates for the 1 lx, 3 lx, and 5 lx contours. Because the 3 lx isoline is used as the representative contour in Figure 2, the same threshold is used for the main descriptor summary. Across the evaluated set, the low-beam 3 lx area ranges from 1171.25 to 4071.75 m^2^ and the high-beam 3 lx area ranges from 2465.50 to 10,476.50 m^2^. The corresponding 3 lx longitudinal reach ranges from 84.6 to 167.6 m for low beam and from 162.6 to 347.1 m for high beam. The 3 lx lateral width ranges from 18.0 to 42.0 m for low beam and from 18.0 to 49.5 m for high beam, while the 3 lx horizontal FOV ranges from 77.4^∘^ to 108.0^∘^ for low beam and from 57.9^∘^ to 106.4^∘^ for high beam. Detailed descriptor ranges are reported in Appendix A.5 and Table A6, and per-system width, reach, and FOV values are listed in Table A7 and Table A8.

#### 3.2.2. Reference Materials and Reflectance Assumptions

Three nominal reflectance levels of 5%, 10%, and 20% are used as reference materials in the benchmark. These values represent difficult, intermediate, and moderately favorable nighttime detection cases and are intended to approximate common pedestrian-clothing reflectances. Several reflectance levels are needed because the same light distribution can yield substantially different signal-to-noise ratios depending on the target surface properties. The benchmark uses measured spectral reflectance curves and evaluates them with a Lambertian reflector model. The headlamp beam pattern defines incident spectral irradiance on the scene, and the Lambertian model converts that irradiance into outgoing spectral radiance at the target surface,

(1) $$L_{o}\left(\lambda \right)=\frac{\rho (\lambda )}{\pi }\;E_{e,\lambda }\left(\lambda \right),$$

which is a controlled first-order approximation for pedestrian fabrics and other diffuse traffic-scene materials. This keeps the primary benchmark values comparable across light distributions because luminance variation is driven primarily by nominal reflectance level rather than by fabric-specific viewing-angle dependence.

To test whether this simplification is adequate for the fabric targets used in the benchmark, measured fabric BRDF data from the public MERL database are used as an uncertainty analysis [25,26]. Nine fabric samples are converted into pointwise luminance scale factors relative to the Lambertian model. For each RoI point, the object surface normal, illumination direction, and camera viewing direction define the BRDF angle. Additional relative azimuth offsets of −20^∘^, −10^∘^, 0^∘^, 10^∘^, and 20^∘^ bound uncertainty in object pose and camera-headlamp separation.

**Figure A6:**
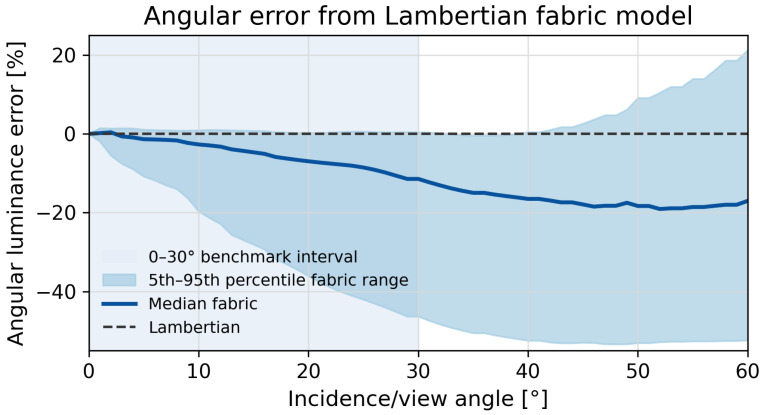
Measured-fabric deviation from Lambertian angular response. The shaded intervals summarize the measured BRDF spread across nine MERL fabric materials after normalization to near-normal response.

**Figure A7:**
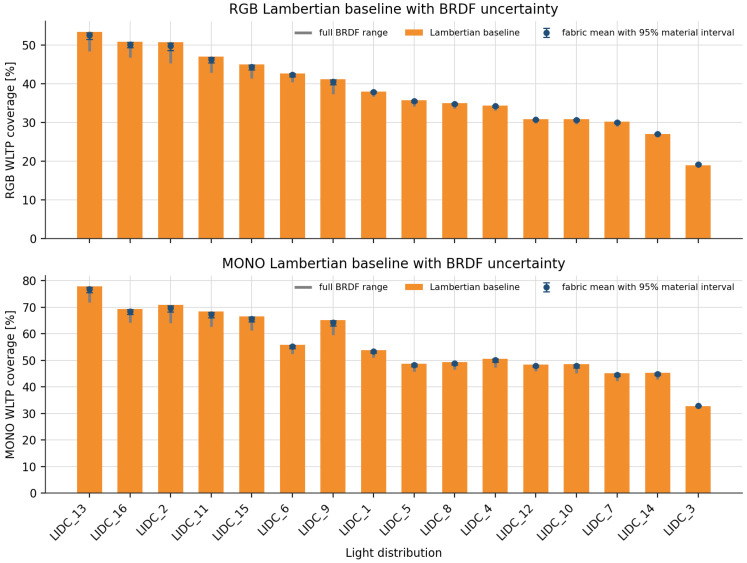
Coverage-level sensitivity to measured fabric BRDFs. Bars show the Lambertian benchmark values used for the main results. Markers and intervals show the measured-fabric BRDF sensitivity around this baseline.

The main benchmark keeps the Lambertian model as the controlled baseline. The BRDF analysis is used only to quantify the uncertainty introduced by this simplification. The detailed angular comparison and coverage-level sensitivity are reported in Appendix A.2, Figure A6 and Figure A7, and Table A2 and Table A3. The measured-fabric analysis changes the mean coverage by less than 1 percentage point for both sensors, and the conservative range remains smaller than the measured coverage spread across the 16 light distributions. This supports the Lambertian model as a controlled first-order approximation for the comparative benchmark.

**Figure 3:**
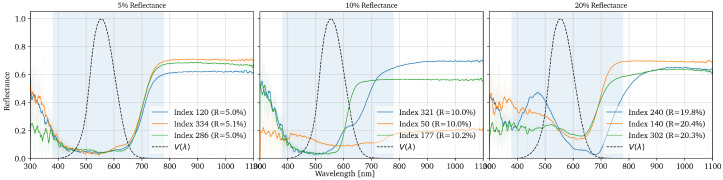
Measured reflectance spectra of the benchmark reference materials. The three nominal reflectance groups represent difficult, intermediate, and easier detection targets for nighttime camera perception; curve colors are identified in the legend.

Figure 3 shows the measured spectral reflectance curves of the three benchmark material groups alongside the standard photopic luminous efficiency function V(λ). The comparison illustrates that the human eye is sensitive only within a spectral band peaked near 555 nm, whereas a monochrome camera sensor can respond across a much wider wavelength range, capturing near-infrared and deep-blue reflectance contributions that are invisible to the human visual system.

### 3.3. Physically Based Camera Model and Validation

The simulation combines headlamp source data and beam-pattern geometry, the diffuse reflector model from Equation (1), a lens transfer function, and an EMVA-style sensor model. The lens maps reflected light to the image plane of the camera and the sensor model converts photon flux into digital signal and noise. The mean signal level is written as

(2) $$\mu_{y}=K\;\left(\mu_{e}+\mu_{D}\right)+y_{0},$$

and the corresponding sensor-domain signal-to-noise ratio is

(3) $$\mathrm{SNR}=\frac{\mu_{e}}{\sqrt{\mu_{e}+\mu_{D}+\sigma_{R}^{2}+\sigma_{Q}^{2}/K^{2}}}.$$

**Figure 4:**
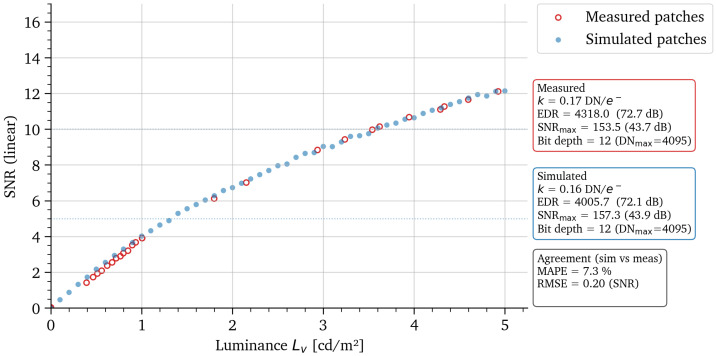
Measured and simulated signal-to-noise ratio agree for the reference camera. The red points show patch-based laboratory measurements, the blue points show pixel-level simulations under matched settings, and the dotted diagonal indicates the 1:1 agreement line. The resulting agreement corresponds to a mean absolute percentage error of 7.3% and a root mean square error of 0.20.

Here, $\mu_{e}$ denotes the mean number of photoelectrons generated by the useful signal, $\mu_{D}$ the dark signal, $\sigma_{R}$ the read noise, $\sigma_{Q}$ the quantization noise, *K* the system gain, and $y_{0}$ the digital offset [27,28,29,30]. This model is necessary because nighttime perception operates near the low-signal noise floor. At small $\mu_{e}$, the denominator is dominated by read and quantization terms, whereas at larger $\mu_{e}$ the system transitions toward the shot-noise-limited regime. Capturing that transition is necessary if headlamp-driven photon allocation is to be translated into perception-relevant signal quality rather than into a purely photometric proxy. The formulation captures the dominant signal-path dependencies, and the relative comparison does not require exhaustive scene-physics fidelity. The camera model is validated against a representative automotive field-test camera by comparing patch-based laboratory measurements with matched pixel-level simulations. Figure 4 summarizes the agreement. The resulting mean absolute percentage error is 7.3%, and the root mean square error is 0.20, confirming that the model serves as a reliable transfer function from photometric input to sensor-domain signal quality. Extensions covering richer material descriptions and atmospheric effects remain future work.

All coverage results in this paper are produced with a fixed baseline camera configuration: 2.5 μm pixel pitch, f/2.8 aperture for the lens system, 20 ms exposure time, Bayer-pattern RGB color filter array, 0.8 peak quantum efficiency, 1.0 e^−^ read noise, 0.1 e^−^/s dark current, and 0.4 DN/e^−^ system gain. This configuration represents a contemporary automotive camera and derives from a field-test instrument whose EMVA-1288 parameters were measured directly. The complete parameter set and the one-factor sweep grid are listed in Appendix A.

### 3.4. Safety-Driven Perception Constraints

The safety and perception constraints are treated as one coupled stage of the benchmark. The region of interest defines where detection matters most in space and time, and the task-derived signal-to-noise map defines how much image quality is required within that region. Coverage is only reported after both constraints are combined.

#### 3.4.1. Safety-Derived Region of Interest

To operate safely at night, a perception system must not only know where to detect objects, but how well they must be detected. The region of interest translates physical traffic dynamics into a spatial priority map that reflects both temporal urgency and object relevance. The goal is to prioritize those regions in the camera image where potential collisions may occur first, ensuring that the perception system allocates sufficient accuracy and robustness to these critical areas. It is derived from vulnerable road-user classes, their typical motion, time-to-collision categories, and reaction urgency. Pedestrians receive the highest weighting because they combine high accident relevance with unpredictable behavior.

Table 2 defines the benchmark’s vulnerable-road-user categories. Table 3 defines the time-to-collision categories used to express urgency. The chosen binning is consistent with published conflict-analysis, warning-timing, and risk-assessment ranges [31,32,33,34,35]. Table 4 adds a braking-based interpretation of urgency and provides an intuitive kinematic link between perception timing and intervention severity.

The importance percentages in these tables define the required detection reliability at each point in the region of interest. An importance of 99.9% dictates that the target must be detected with 99.9% of the neural network’s peak average precision, which in turn demands the highest signal-to-noise ratio from the camera system. Combining the deceleration categories with the time-to-collision framework maps physical traffic dynamics onto a continuous scale of perceptual priority.

For a given speed *v* and reaction time $t_{\mathrm{react}}$, the additional distance traveled before any intervention is

(4) $$d_{\mathrm{react}}=v\;t_{\mathrm{react}},$$

and the standard stopping-distance formulation provides a physically intuitive reference,

(5) $$s_{\mathrm{total}}=v\;t_{\mathrm{react}}+\frac{v^{2}}{2a_{\mathrm{brake}}}.$$

**Figure 5:**
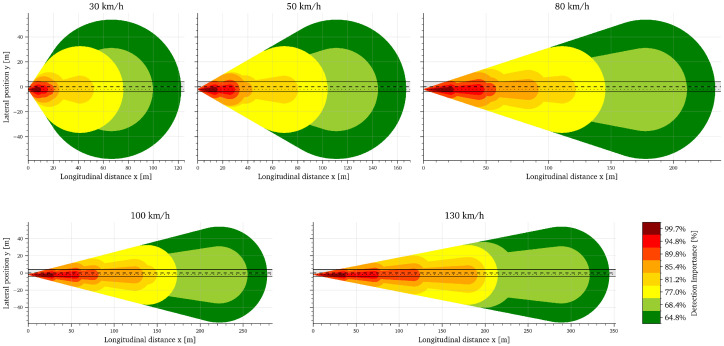
Region-of-interest maps defining where and how well the system must detect objects across the five benchmark speeds. Warmer colors indicate regions with the highest temporal urgency, which consequently demand the highest signal-to-noise ratio from the headlamp-camera system to ensure reliable detection. Dashed lines denote the road reference geometry.

Because multiple potential VRU trajectories and reaction scenarios overlap in the driving space, the spatial priority map is constructed under a worst-case assumption. Each coordinate in the region of interest therefore inherits the maximum importance value produced by any admissible trajectory and braking scenario at that location. This ensures that the benchmark does not average away rare but safety-critical interactions. Every point is instead evaluated according to the most urgent conflict that could plausibly occur there. In the benchmark, the time-to-collision categories remain fixed, but their spatial positions move farther from the vehicle as speed increases. The resulting region of interest therefore extends strongly in the longitudinal direction at higher speed while preserving lateral support for possible side entry of vulnerable road users. The benchmark evaluates 30, 50, 80, 100, and 130 km/h because these operating points span urban, interurban, and highway use cases. The resulting maps are shown in Figure 5.

**Figure 6:**
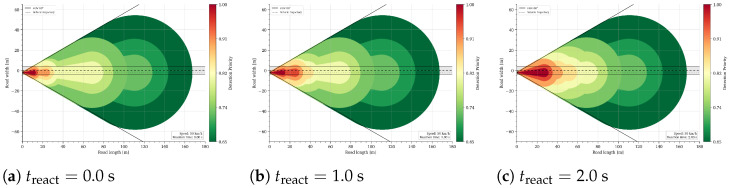
Reaction time shifts the region of interest at 50 km/h. Longer reaction times enlarge the high-priority longitudinal region because the detection task must be solved earlier in space to maintain the same temporal margin. Dashed lines denote the road reference geometry.

Reaction time provides the link from scene urgency to system latency. The parameter $t_{\mathrm{react}}$ is treated as a conservative aggregate perception-to-actuation latency covering sensing, software, decision, and execution delay in assisted and automated-driving stacks, not as a purely human reflex quantity. Human driver perception-brake times for unexpected events range from 0.8 to 1.5 s depending on expectancy and workload [36]. Automated driving stacks likewise introduce finite pipeline latency: measured end-to-end delays from perception to control range from approximately 150 ms up to 500 ms depending on architecture and communication design, while aspirational targets for safe automated response are below 100 ms [37,38,39]. Increasing the latency budget shifts the critical zone farther away from the vehicle, because the same time-to-collision threshold must be satisfied at a larger physical distance. The 1.0 s baseline therefore covers the upper range of human reaction latency and provides a conservative systems-level margin for ADAS pipelines that must account for multi-frame confirmation, communication overhead, and actuator response delay. The shift is illustrated in Figure 6.

#### 3.4.2. Vision-Task-Derived Signal-to-Noise Requirements

Object recognition is the foundational perception primitive for ADAS and automated-driving systems: every higher-order function, from motion forecasting to path planning, presupposes reliable initial identification of relevant scene objects. The present benchmark derives minimum signal-to-noise requirements directly from a controlled object-recognition study, treating recognition performance as the first-principles link between sensor-domain image quality and downstream driving safety. Prior literature discusses nighttime detection in terms of broad fixed thresholds, commonly around linear signal-to-noise ratios of 5 to 10 [21,22,23,24]. Those values serve as a useful baseline but cannot distinguish object scale, target difficulty, or safety relevance. The benchmark replaces this single global threshold with required signal-to-noise values tied to defined detection-quality targets and mapped spatially across the region of interest.

Six established neural-network architectures, including convolutional and transformer-based models, are evaluated under progressively reduced image quality. Image quality is controlled by adding zero-mean Gaussian noise to the evaluation images, with the noise standard deviation scaled to yield a target linear SNR level *s*. During fine-tuning, 10% of the training samples are corrupted with Gaussian noise, and the training SNR is sampled uniformly from −10 dB to 40 dB. The subsequent SNR sweep uses the same corruption family at fixed SNR levels from −10 dB to 40 dB in 1 dB steps. The model set covers a broad design space and prevents the benchmark from encoding the bias of a single architecture.

The training dataset is constructed from Cityscapes [40] object crops and contains 14,989 pedestrian samples together with 91,557 samples from other object classes. The validation split contains 6424 pedestrian samples and 39,260 non-pedestrian objects. All models are trained under matched augmentation and sampling conditions so that differences in robustness can be attributed primarily to architecture rather than to training setup. Weighted sampling is used to counter class imbalance, and the final signal-to-noise requirement curves are derived from the average precision behavior aggregated across the six networks. The ensemble includes ResNet [15], MobileNetV3 [16], ViT [17], EfficientNetV2 [18], SwinV2 [19], and ConvNeXt V2 [20], so the resulting requirement map is not tied to one backbone family. The evaluated architectures are summarized in Table 5.

**Figure 7:**
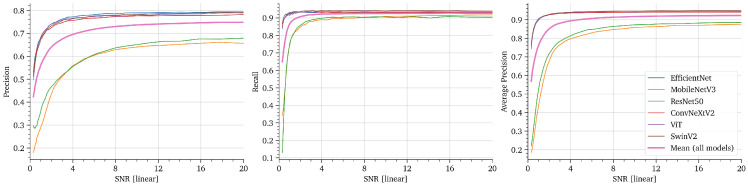
Ensemble detection metrics as functions of linear signal-to-noise ratio. The precision, recall, and average-precision trends across the six-model ensemble provide the task-performance basis for the required-SNR curves used in the benchmark.

The core evaluation is shown in Figure 7. The aggregate all-object curve is the relevant reference for the present paper because it captures the robustness trend across architectures without overloading the main text with secondary breakdowns. Detection performance remains comparatively stable in the high-SNR regime and then drops much more steeply once the linear signal-to-noise ratio approaches approximately five. This shared transition zone is more important for the benchmark than the precise ranking between individual networks.

**Figure 8:**
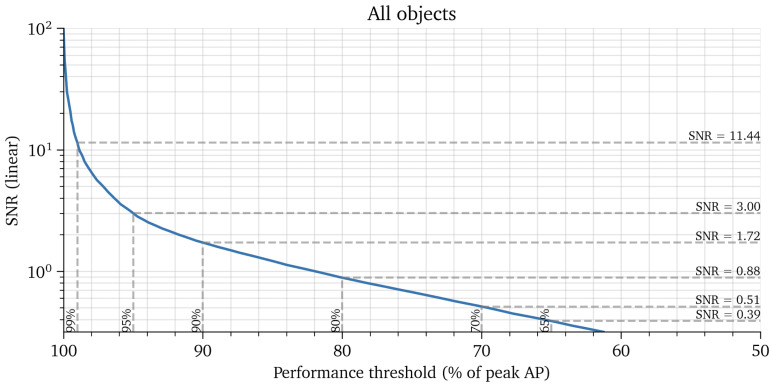
Required signal-to-noise ratio from the all-objects evaluation. The curve provides the perception threshold used in the benchmark for the aggregate object set.

For each network m∈{1,…,6}, the benchmark defines the ensemble average-precision curve

(6) $$\bar{\mathrm{AP}}\left(s\right)=\frac{1}{6}\sum_{m=1}^{6}{\mathrm{AP}}_{m}\left(s\right),$$

where *s* denotes the linear input signal-to-noise ratio used during the controlled corruption study. The required signal-to-noise ratio for a target fraction α∈(0,1] of the peak ensemble performance is then defined by the inverse rule

(7) $${\mathrm{SNR}}_{\mathrm{req}}\left(\alpha \right)=\inf\left\{s:\bar{\mathrm{AP}}\left(s\right)\geq \alpha \;\max_{u}\bar{\mathrm{AP}}\left(u\right)\right\},$$

implemented with monotone interpolation of the measured ensemble curve. The resulting required-SNR curve is shown in Figure 8.

The final requirement map is obtained by assigning each point in the region of interest a target fraction of peak average precision according to its safety importance. Using the normalized RoI importance field I(x)∈[0,1],

(8) $${\mathrm{SNR}}_{\mathrm{req}}\left(x\right)={\mathrm{SNR}}_{\mathrm{req}}\left(I(x)\right).$$

High-priority regions therefore require higher signal quality than peripheral planning zones.

### 3.5. System Evaluation Through Coverage

The final benchmark integrates the validated camera model, the light-distribution dataset, the safety-derived region of interest, and the representative material reflectances. Coverage is defined as the fraction of the region of interest in which the simulated signal-to-noise ratio exceeds the locally required threshold. The metric can therefore be interpreted as the portion of the relevant scene that is sufficiently illuminated for the target perception task.

For one light distribution *l*, one speed *v*, and one reflectance level ρ, the local success indicator is

(9) $$\chi_{l,v,\rho }\left(x\right)=\left\{\begin{array}{cc} 1, & {\mathrm{SNR}}_{l,v,\rho }\left(x\right)\geq {\mathrm{SNR}}_{\mathrm{req}}\left(x\right),\\ 0, & \mathrm{otherwise}, \end{array}\right.$$

and the corresponding region-of-interest coverage is

(10) $$C_{l,v,\rho }=\frac{1}{|\Omega_{v}|}\sum_{x\in \Omega_{v}}\chi_{l,v,\rho }\left(x\right),$$

where $\Omega_{v}$ denotes the discrete RoI for the selected speed. Coverage is first averaged across the three reflectance levels for each speed,

(11) $${\bar{C}}_{l,v}=\frac{1}{\left|R\right|}\sum_{\rho \in R}C_{l,v,\rho }.$$

The primary comparison score is then computed as a WLTP moving-time weighted average over the five speed scenarios,

(12) $$C_{l}^{\mathrm{WLTP}}=\sum_{v\in V}w_{v}\;{\bar{C}}_{l,v},$$

with V={30,50,80,100,130} km/h. The weights are derived from the Class 3b Worldwide harmonized Light vehicles Test Cycle (WLTC) velocity trace used within the WLTP by excluding standstill samples and binning the remaining 1 s samples into the benchmark speed scenarios [41,42]. The resulting weights are listed in Table 6. The arithmetic mean over speed remains a reference value for comparison with the WLTP-weighted score.

**Figure 9:**
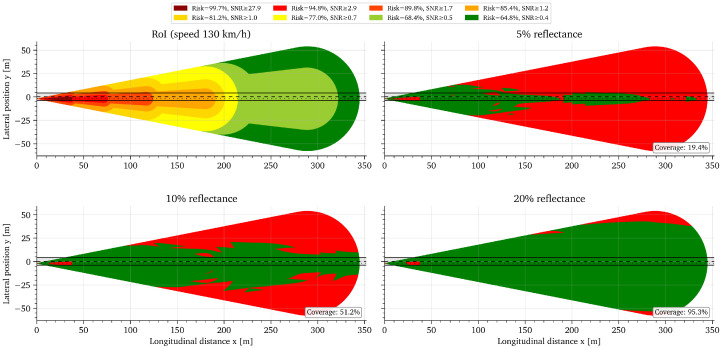
Example benchmark output for one light distribution at 130 km/h. Green regions satisfy the required signal-to-noise ratio, whereas red regions indicate insufficient signal quality. Dashed and dotted overlays indicate the road and grid reference geometry.

An example pass/fail coverage map for one light distribution is shown in Figure 9.

## 4. Results

The results report the comparative coverage ranking across all 16 light distributions, the sensitivity to the reaction-time assumption, the sensor-parameter hierarchy, and the difference between monochrome and color sensing.

### 4.1. Comparative Evaluation of 16 Light Distributions

**Figure 10:**
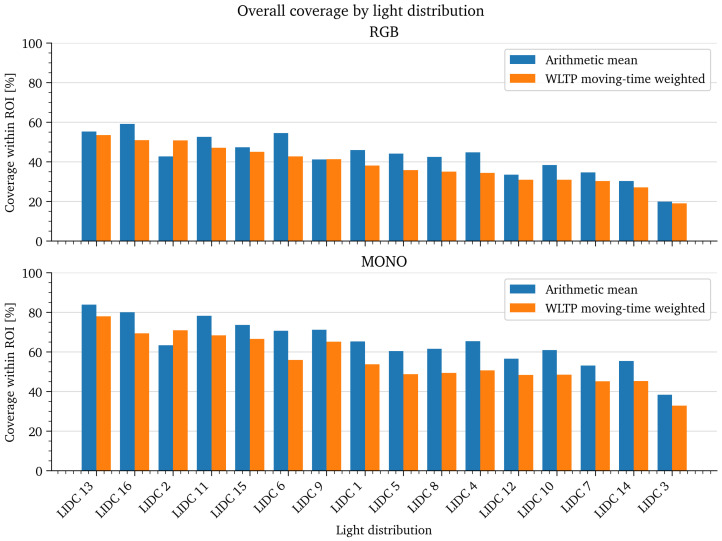
Overall coverage for the evaluated light distributions. The WLTP-weighted score is the primary benchmark value and the arithmetic mean is shown as a reference.

The WLTP-weighted benchmark reveals a large spread across the 16 evaluated light distributions. For the baseline RGB camera, the strongest system reaches 53.48% coverage, whereas the weakest reaches 18.95%. This corresponds to a factor of 2.82 and shows that beam design is a primary determinant of camera-relevant nighttime signal quality, as shown in Figure 10.

The separation remains visible when the arithmetic speed average is shown as a reference. For RGB sensing, the arithmetic mean spans from 19.82% to 59.14%. For monochrome sensing, the arithmetic mean spans from 38.31% to 83.85%. The ranking therefore reflects systematic beam-pattern differences rather than only the chosen speed-aggregation rule, as shown in Figure 10 and illustrated by the isoline examples in Figure 2. The aggregate values are summarized in Table 7.

**Figure 11:**
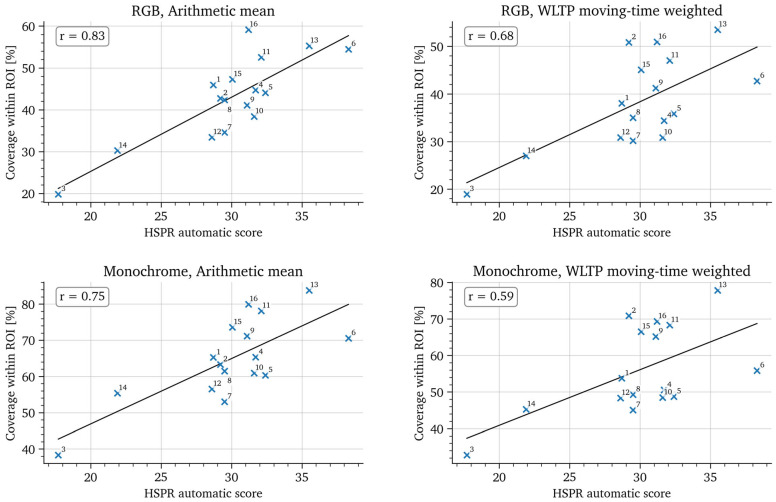
Correlation between benchmark coverage and headlamp safety performance rating. The bluemarkers show valid HSPR entries, and numeric point labels identify the anonymized light-distribution categories. The WLTP-weighted benchmark is positively correlated with the human-centric HSPR score.

To position the proposed benchmark relative to an established automotive lighting metric, the coverage values are also compared with the automatic-operation headlamp safety performance rating listed in Table 1. The WLTP-weighted coverage score correlates positively with HSPR, with Pearson’s *r* = 0.68 for the RGB configuration. The arithmetic reference gives *r* = 0.83, indicating that the speed-weighting rule changes the numerical alignment with HSPR. The qualitative conclusion remains unchanged because the human-centric rating and camera-oriented coverage are partially aligned but not interchangeable. The correlation is shown in Figure 11.

### 4.2. Reaction-Time Sensitivity

The baseline RoI uses $t_{\mathrm{react}}=1.0$ s as a conservative systems-level latency margin. To test the influence of this assumption, the final coverage score is recomputed for $t_{\mathrm{react}}=0.0$, 0.1, 0.5, 1.0, and 2.0 s. The intermediate 0.5 s setting changes the full-RoI WLTP mean only slightly relative to the 1.0 s baseline, as shown in Table 8. The RGB mean increases by 0.276 percentage points and the monochrome mean increases by 0.135 percentage points. The 2.0 s stress case produces the larger effect and reduces the RGB mean by 2.210 percentage points and the monochrome mean by 1.634 percentage points.

The small full-RoI sensitivity follows from the metric definition. The full-RoI average contains many lower-risk cells and many cells whose signal-to-noise ratio remains clearly above or below the local decision threshold when the reaction-time parameter changes. Only the cells that move close to the SNR decision boundary alter their pass/fail state. Appendix A.4 reports a complementary high-risk analysis restricted to the three largest RoI risk bands, where the reaction-time effect is more visible.

### 4.3. Camera-Parameter Hierarchy and Design Trade-Offs

The one-factor parameter sweeps show a clear hierarchy of influence. Pixel size of the camera sensor has the strongest effect on coverage, followed by exposure time and aperture. Read noise, system gain, and peak quantum efficiency have smaller but still visible impact, whereas dark current is negligible within the explored range. Across all sweeps, however, the ranking gap between good and weak light distributions remains visible. Lighting quality is therefore persistent rather than replaceable by minor sensor tuning.

**Figure 12:**
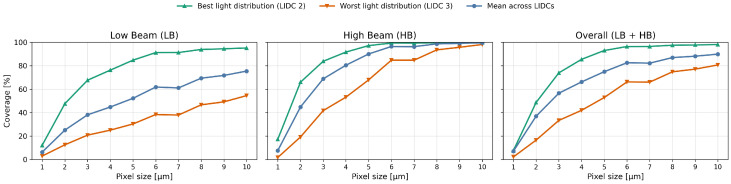
Coverage as a function of pixel size. The parameter range is large, but the light-distribution-dependent separation remains visible over the full sweep. The three line styles show the best, worst, and mean coverage across the evaluated light distributions.

**Figure 13:**
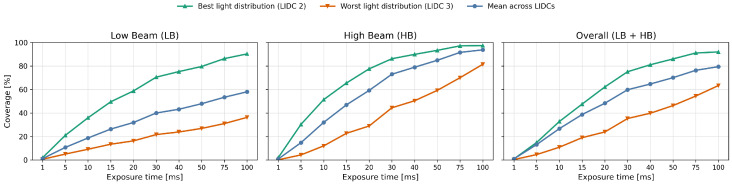
Coverage as a function of exposure time. Exposure provides a strong sensor-side lever, but it does not remove the performance spread caused by the light distribution. The three line styles show the best, worst, and mean coverage across the evaluated light distributions.

Figure 12 highlights the dominant influence of pixel size, while Figure 13 shows that longer exposure improves coverage but introduces a design trade-off with dynamic imaging constraints. In both cases, a favorable light distribution shifts the achievable operating point markedly upward.

The engineering meaning of this ranking is direct. Enlarging pixel size increases the photonic input per pixel, and longer exposure increases the integrated photon count, so both strongly improve signal-to-noise ratio across the region of interest. Aperture acts in the same direction and therefore forms the third major hardware lever. By contrast, read noise, gain, and peak quantum efficiency offer smaller improvements within the explored range, and dark current is effectively non-limiting for the short automotive exposures considered here. All remaining one-factor sweep results are provided in Appendix A.1.

At constant perception coverage, beam quality can partially substitute for sensor sensitivity. In the pixel-size sweep, the strongest low-beam distribution reaches a coverage at a 4 μm pixel pitch that the weakest distribution only attains near 10 μm, implying that improved beam placement can reduce the required pixel pitch by roughly 60% while maintaining comparable benchmark performance. The same pattern appears in the exposure sweep, where favorable light distributions reach equivalent operating points at materially shorter integration times and therefore with less motion-blur risk. The aperture sweep reinforces the same point: a premium distribution at a wide aperture remains far ahead of a poor distribution even when the latter is granted aggressive sensor-side compensation, so the benchmark gain available from beam optimization is comparable to or larger than major hardware changes on the camera side.

The benchmark gap caused by beam placement remains visible across all sensor sweeps. Illumination quality is therefore a primary design factor in the same signal path as pixel size, exposure time, aperture, and sensor noise.

### 4.4. Monochrome Versus Color Sensing

**Figure 14:**
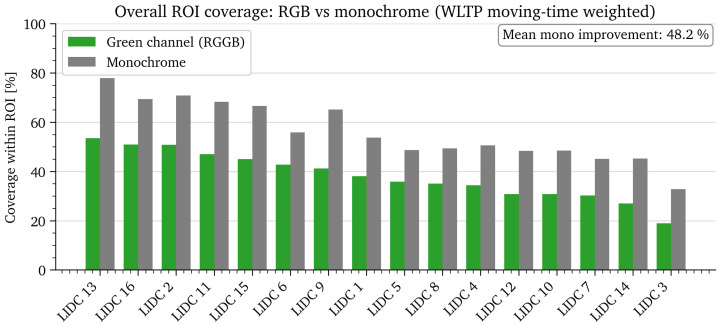
Overall coverage for color and monochrome sensing. The monochrome configuration consistently yields higher coverage across the evaluated light distributions.

The benchmark also shows a consistent sensitivity advantage for monochrome imaging over a conventional Bayer-pattern color sensor. For this comparison, the sensor architecture, pixel pitch, lens model, aperture, exposure time, spectral quantum-efficiency curve, and EMVA noise parameters remain identical. Only the spectral transmission introduced by the color filter array changes, and the channel transmission is set to unity for the monochrome configuration. Averaged across the light distributions with WLTP weighting, the monochrome configuration improves mean coverage from 38.277% to 56.014%, corresponding to an improvement of 46.3%, as shown in Figure 14. This result is secondary to the beam-pattern ranking and parameter hierarchy, but it remains relevant for nighttime perception tasks in which signal quality and spatial resolution are more critical than color information.

## 5. Discussion

For the RGB configuration, the WLTP-weighted benchmark separates the strongest and weakest light distributions by a factor of 2.82, with coverage ranging from 18.95% to 53.48%. This spread is large compared with the uncertainty analyses and confirms that the road illumination pattern is a primary design variable for nighttime camera perception.

The parameter sweeps resolve that finding into an engineering hierarchy. Larger pixels, longer exposure times, and wider camera lens apertures improve coverage strongly, but none of these changes collapse the gap between good and poor light distributions. Sensor tuning alone is therefore an inefficient compensation for an unfavorable beam pattern, and joint optimization of illumination and sensing is a more effective design approach. The pixel-size and aperture equivalences are large enough that beam optimization can offset hardware changes that would otherwise require materially larger sensors or optics.

The positive correlation with headlamp safety performance rating confirms partial alignment between human-centric and camera-centric illumination assessment, but the two metrics are not interchangeable. Human-centric ratings quantify visibility outcomes for drivers. The proposed benchmark evaluates how available irradiance translates into camera signal quality in safety-critical scene regions. That distinction motivates the dedicated perception-oriented benchmark.

The present model is limited to the core signal-path components. The benchmark uses a validated model built from measured spectra and beam patterns, a controlled diffuse-reflectance abstraction, a straightforward imaging-transfer model, and an EMVA-based camera description. This makes the comparison interpretable, but it does not cover the full range of physical scene effects encountered in real traffic.

The measured-fabric BRDF sensitivity changes the mean WLTP coverage by −0.35 percentage points for RGB and −0.68 percentage points for the monochrome configuration in the nominal relative-azimuth case, with conservative material and azimuth ranges of −5.47 to 0.78 percentage points for RGB and −6.81 to 0.29 percentage points for the monochrome configuration. These bounds are smaller than the 34.53 percentage point RGB spread and 45.07 percentage point monochrome spread across the light-distribution dataset.

**Figure A8:**
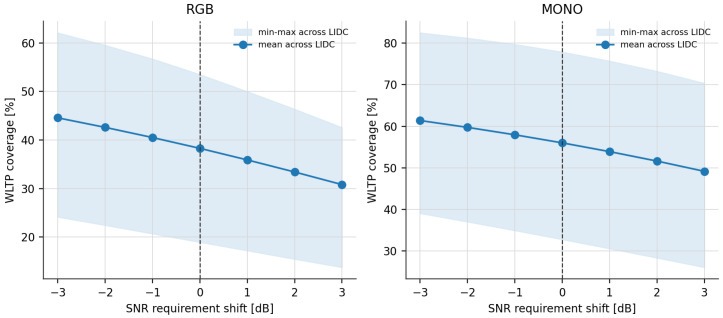
Coverage sensitivity to shifts of the required SNR curve. Negative shifts represent less conservative SNR requirements, and positive shifts represent more conservative requirements.

The SNR-to-performance mapping uses models trained with Gaussian-noise augmentation, but Gaussian corruption remains an approximation of physical camera noise because real sensors also include signal-dependent shot noise, read-noise structure, and spatial or spectral nonuniformity. The use of noise augmentation reduces the clean-training brittleness mechanism, while any remaining bias from limited noise diversity is expected to act mainly by making the required-SNR curve conservative relative to more robustly trained detectors. The reported coverage values are therefore interpreted as lower-bound estimates with respect to such detectors. Shifting the required SNR curve by ±3 dB changes the mean WLTP coverage by +6.29 and −7.47 percentage points for RGB and by +5.38 and −6.85 percentage points for the monochrome configuration. The full shift analysis is reported in Appendix A.3, Figure A8 and Table A4. The best and worst light distributions remain unchanged in these SNR-shift tests.

The raw headlamp IES files remain proprietary, so reproducibility is supported through the released scripts, benchmark summary tables, and non-reconstructive photometric descriptors rather than by publishing the full measured distributions. On the perception side, the benchmark logic extends from recognition toward tracking and segmentation in dynamic scenes, enabling evaluation not only of whether critical objects are seen but of whether they remain reliably localized and behaviorally interpretable over time.

## 6. Conclusions

With WLTP moving-time aggregation, baseline RGB coverage ranges from 18.95% to 53.48% across the 16 measured systems, corresponding to a factor of 2.82 between the weakest and strongest light distribution. This range identifies beam placement as a primary variable for automotive camera perception at night.

The parameter sweeps translate that separation into engineering terms. Better beam placement delivers comparable benchmark performance with roughly 60% smaller pixel pitch than the weakest distribution, corresponding to an 84% reduction in pixel area, or with materially shorter exposure times. The gain from a strong light distribution rivals or exceeds hardware improvements such as optics with greater numerical apertures. Perception reliability at night should not be treated as a sensor-hardware or algorithm problem alone, and lighting and sensing must be designed as one system.

## Figures and Tables

**Table 1:** Evaluated light-distribution categories and headlamp safety ratings. The table lists the 16 light-distribution identifier categories together with the corresponding headlamp safety performance rating for low-beam, high-beam, and automatic operation. Automatic operation combines low-beam and automatically activated high-beam use into one value.

LIDC	Low Beam		High Beam		Automatic	
	HSPR	Rating	HSPR	Rating	HSPR	Rating
LIDC 1	3.52	Excellent	3.46	Excellent	28.7	Advanced
LIDC 2	5.52	Premium+	−0.14	Standard	29.2	Advanced
LIDC 3	1.39	Good	0.18	Standard	17.7	Good
LIDC 4	4.17	Premium	4.23	Premium	31.7	Excellent
LIDC 5	4.33	Premium	4.36	Premium	32.4	Excellent
LIDC 6	5.83	Premium+	5.44	Premium+	38.3	Excellent
LIDC 7	4.14	Premium	2.81	Advanced	29.5	Excellent
LIDC 8	4.14	Premium	2.81	Advanced	29.5	Excellent
LIDC 9	5.02	Premium+	2.13	Advanced	31.1	Excellent
LIDC 10	3.99	Premium	4.48	Premium	31.6	Excellent
LIDC 11	5.02	Premium+	2.79	Advanced	32.1	Excellent
LIDC 12	4.01	Premium	2.39	Advanced	28.6	Advanced
LIDC 13	5.88	Premium+	3.45	Excellent	35.5	Excellent
LIDC 14	2.39	Advanced	1.08	Good	21.9	Advanced
LIDC 15	4.33	Premium	3.17	Excellent	30.1	Excellent
LIDC 16	3.96	Premium	4.27	Premium	31.2	Excellent

**Table 2:** Nominal vulnerable road-user classes used in the region of interest. The table lists the typical speeds and relative importance values used to derive the region of interest.

VRU	Speed	Importance
Pedestrian	8 km/h	99.9%
Runner or animal	15 km/h	95%
Cyclist or e-scooter	25 km/h	90%

**Table 3:** Situation categories by time to collision and relative importance. The table defines the urgency bins that determine how safety relevance varies with the available time headroom.

Situation	TTC min	TTC max	Importance
Imminent collision	0 s	1 s	99.9%
Critical TTC	>1 s	2 s	95%
Timely response	>2 s	3 s	90%
Look-ahead or planning	>3 s	5 s	80%

**Table 4:** Braking profiles expressed as deceleration and relative importance. The table provides a braking-based interpretation of urgency for the region-of-interest weighting.

Braking Profile	Deceleration	Importance
Emergency braking	8 $m/s^{2}$	99.9%
Normal braking	4 $m/s^{2}$	95%
Comfort braking	2 $m/s^{2}$	90%

**Table 5:** Vision architectures used to derive the benchmark signal-to-noise requirements. The model set covers convolutional and transformer-based architectures to reduce bias toward a single backbone family.

Network	Year	Type	Parameters [M]
ResNet-50D	2015	CNN	25.6
MobileNetV3-Large	2019	CNN	5.4
ViT-Base (16 × 16)	2020	Transformer	86.6
EfficientNetV2-S	2021	CNN	21.5
SwinV2-Tiny	2022	Transformer	28.3
ConvNeXt V2 Nano	2023	CNN	15.6

**Table 6:** WLTP moving-time weights used for coverage aggregation. Standstill samples are excluded because the benchmark does not define a 0 km/h RoI.

Benchmark Speed	WLTC Speed Bin	Weight
30 km/h	0<v<40 km/h	0.4080
50 km/h	40≤v<65 km/h	0.2797
80 km/h	65≤v<90 km/h	0.1367
100 km/h	90≤v<115 km/h	0.1047
130 km/h	v≥115 km/h	0.0709

**Table 7:** Aggregated benchmark results for the evaluated light distributions. The table reports the minimum, maximum, and mean coverage across all 16 light distributions for both aggregation rules.

Sensor	Average	Min [%]	Max [%]	Mean [%]	Factor	Best/Worst
RGB	Arithmetic	19.817	59.143	42.886	2.98	LIDC 16/LIDC 3
RGB	WLTP	18.951	53.482	38.277	2.82	LIDC 13/LIDC 3
Monochrome	Arithmetic	38.306	83.847	64.824	2.19	LIDC 13/LIDC 3
Monochrome	WLTP	32.794	77.862	56.014	2.37	LIDC 13/LIDC 3

**Table 8:** Reaction-time sensitivity of the WLTP-weighted coverage score. The 1.0 s setting is the baseline used for the main benchmark.

Sensor	$t_{\mathrm{react}}$ [s]	WLTP Mean [%]	Difference to 1.0 s [pp]
RGB	0.0	38.895	0.618
RGB	0.1	38.831	0.554
RGB	0.5	38.553	0.276
RGB	1.0	38.277	0.000
RGB	2.0	36.067	−2.210
Monochrome	0.0	56.338	0.324
Monochrome	0.1	56.299	0.285
Monochrome	0.5	56.149	0.135
Monochrome	1.0	56.014	0.000
Monochrome	2.0	54.380	−1.634

**Figure A1:**
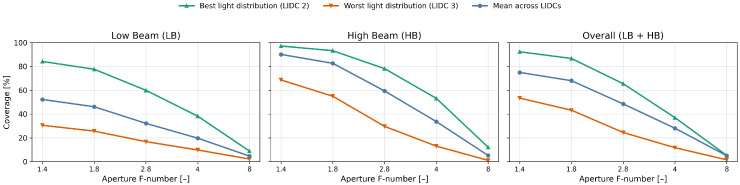
Coverage as a function of aperture. Wider apertures improve the photon budget and therefore increase coverage, but the separation between favorable and weak light distributions remains visible across the full range.

**Figure A2:**
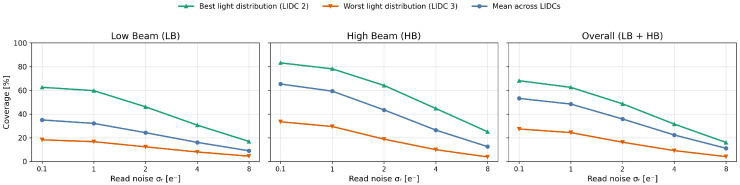
Coverage as a function of read noise. Read-noise reduction improves coverage, but the effect remains smaller than the dominant photon-budget levers highlighted in the main text.

**Figure A3:**
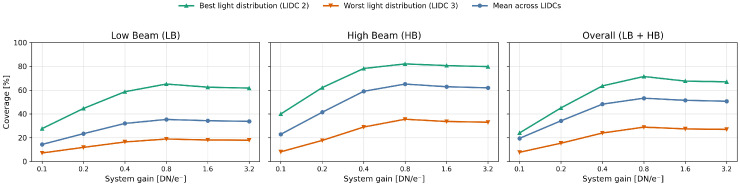
Coverage as a function of system gain. Gain changes the coverage level only moderately over the explored range and does not remove the beam-pattern-dependent separation.

**Figure A4:**
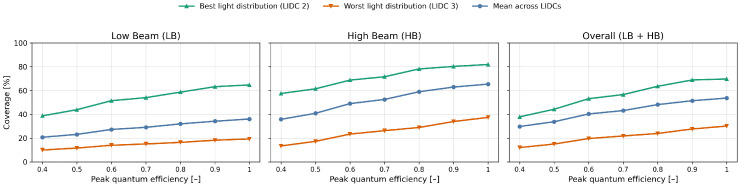
Coverage as a function of peak quantum efficiency. Higher peak quantum efficiency produces a moderate upward shift in coverage across the evaluated light distributions.

**Figure A5:**
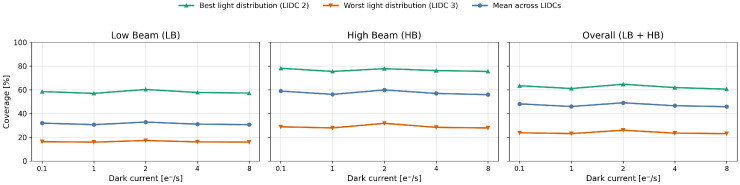
Coverage as a function of dark current. Dark current remains nearly negligible within the short-exposure automotive regime considered here.

**Figure A9:**
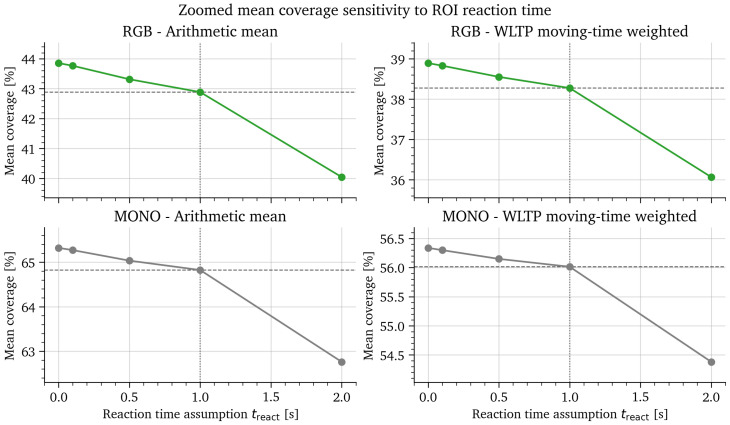
Zoomed full-RoI reaction-time sensitivity. The 0.5 s setting changes the WLTP-weighted mean by less than 0.3 percentage points for RGB and less than 0.2 percentage points for the monochrome configuration. The dashed line marks the 1.0 s baseline.

**Figure A10:**
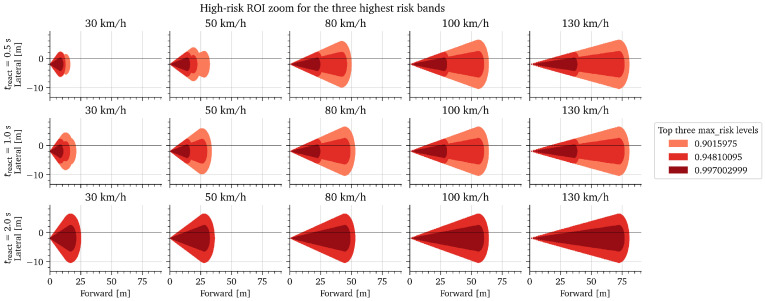
High-risk RoI subset used for the reaction-time sensitivity check. The plot shows only the three largest discrete RoI risk levels.

**Figure A11:**
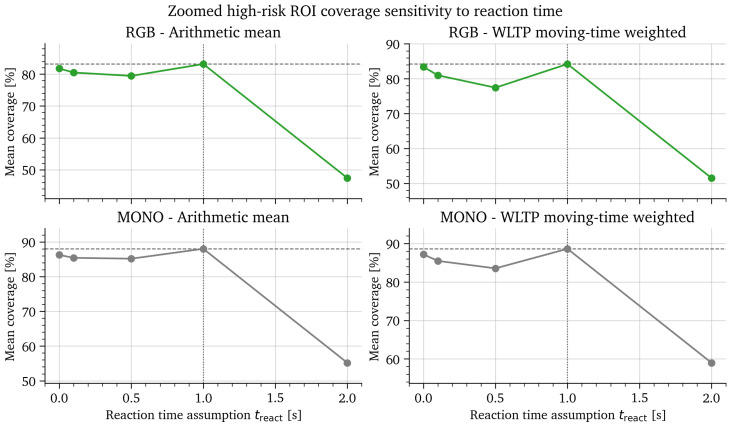
Reaction-time sensitivity in the highest RoI risk bands. The high-risk subset shows a stronger dependence on the reaction-time assumption than the full-RoI average.

**Figure A12:**
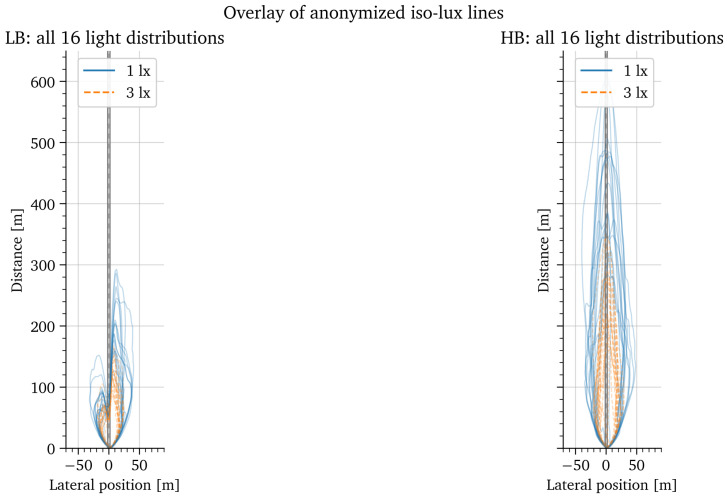
Overlay of anonymized iso-lux lines for all light distributions. The plot compares the 1 lx and 3 lx contours for low beam and high beam without exposing the raw IES files.

**Figure A13:**
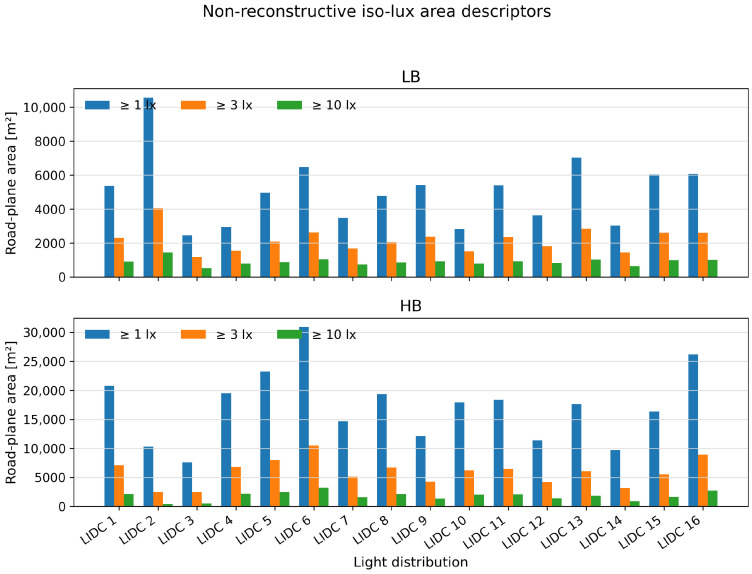
Iso-lux area of the evaluated light distributions. The bars report non-reconstructive area descriptors for the anonymized light-distribution set.

**Figure A14:**
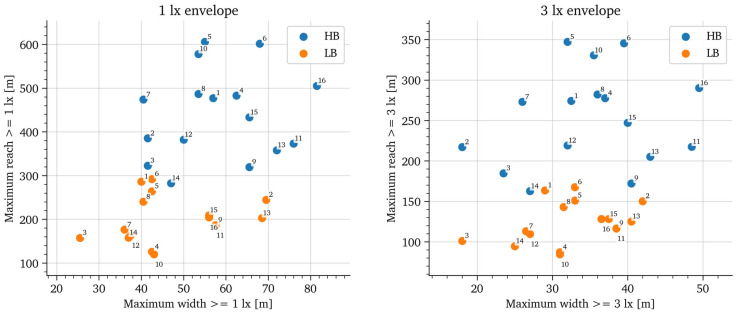
Road-plane reach and width of the evaluated light distributions. The descriptors quantify longitudinal and lateral spread without publishing the raw beam patterns; numeric labels identify the anonymized light-distribution categories.

## Data Availability

The raw headlamp IES light-distribution files are proprietary and cannot be made publicly available. Anonymized, non-reconstructive photometric descriptor tables for all 16 light distributions are provided in the appendix and include iso-lux area, longitudinal reach, lateral width, field-of-view estimates, and angular spread descriptors. The non-proprietary supplementary data package contains the benchmark aggregation scripts, WLTP speed weights, benchmark summary tables, reaction-time sensitivity tables, BRDF uncertainty tables, SNR uncertainty tables, and coverage-summary CSV outputs.

## Abbreviations

The following abbreviations are used in this manuscript:

ADAS	Advanced driver-assistance systems
ADB	Adaptive Driving Beam
AP	Average precision
BRDF	Bidirectional Reflectance Distribution Function
EMVA	European Machine Vision Association
FOV	Field of view
HSPR	Headlamp Safety Performance Rating
IES	Illuminating Engineering Society
LIDC	Light Intensity Distribution Curve
MAPE	Mean absolute percentage error
QE	Quantum efficiency
RGB	Red, Green, Blue
RMSE	Root mean square error
RoI	Region of interest
SNR	Signal-to-noise ratio
TTC	Time to collision
VRU	Vulnerable road user
WLTP	Worldwide harmonized Light-duty vehicles Test Procedure
WLTC	Worldwide harmonized Light vehicles Test Cycle

## Appendix A. Detailed Baseline and Sweep Grid

Table A1 gives the full baseline-and-sweep definition used for the one-factor parameter analysis. In each sweep, only the listed parameter varies while all remaining parameters remain fixed at the baseline value.

**Table A1:** Detailed baseline and sweep grids for the one-factor camera-parameter analysis. The table reports the baseline setting and the varied parameter levels used in the one-factor sensitivity study.

Parameter	Symbol [Unit]	Baseline	Sweep Levels
Pixel size	*p* [μm]	2.5	1–10 μm in integer steps
Aperture (F-number)	*N* [–]	2.8	{1.4, 1.8, 2.8, 4.0, 8.0}
Read noise	$\sigma_{r}$ [e^−^]	1.0	{0.1, 1, 2, 4, 8}
System gain	*K* [DN/$e^{-}$]	0.4	{0.1, 0.2, 0.4, 0.8, 1.6, 3.2}
Peak quantum efficiency	${\mathrm{QE}}_{\max}$ [–]	0.8	{0.4, 0.5, 0.6, 0.7, 0.8, 0.9, 1.0}
Dark current	$i_{\mathrm{dark}}$ [$e^{-}$/s]	0.1	{0.1, 1, 2, 4, 8}
Optical transmission	$\tau_{\mathrm{opt}}$ [–]	0.8	fixed
Maximum code value	${\mathrm{DN}}_{\max}$ [DN]	4096	fixed
Color filter array	–	RGB	fixed
Exposure time	$t_{\exp}$ [ms]	20	{1, 5, 10, 15, 20, 30, 40, 50, 75, 100}
Light distributions	–	LIDC 1–16	reported as best, worst, mean

### Appendix A.1. Additional One-Factor Sweep Results

The main text highlights the pixel-size and exposure-time sweeps because they provide the clearest sensor-side interpretation of the benchmark. For completeness, the remaining one-factor results are reported here. Together with the main-text sweeps, Figure A1, Figure A2, Figure A3, Figure A4 and Figure A5 complete the appendix-level overview of how aperture, read noise, system gain, peak quantum efficiency, and dark current influence coverage.

### Appendix A.2. Fabric BRDF Check for the Lambertian Assumption

The Lambertian reflector assumption is tested with nine measured fabric BRDFs from the MERL database. For the angular-only comparison, each material response is normalized to its near-normal value, so the comparison isolates angular response instead of absolute fabric albedo. Figure A6 shows that most fabric samples remain close to the Lambertian reference in the angular range used by the benchmark.

**Table A2:** Fabric BRDF deviation from the Lambertian reference. The benchmark-relevant interval is between 0 and 30^∘^.

Angular Interval	$k_{\min}$	$k_{\mathrm{median}}$	$k_{\max}$	Median abs. Error [%]	Max abs. Error [%]
0 to 30^∘^	0.436	0.975	1.023	4.69	56.39
30 to 60^∘^	0.353	0.845	1.247	17.50	64.73

Figure A7 translates the same measured-fabric BRDF set to coverage-level uncertainty. The bars show the Lambertian benchmark values used for the main results. Markers and intervals show the measured-fabric BRDF sensitivity around this baseline. The central estimate uses the mean of the nine fabrics in the nominal relative-azimuth case. The interval is a 95% measured-fabric bootstrap interval over these nine materials, and the conservative range includes all tested relative azimuth offsets.

**Table A3:** Coverage-level effect of the measured-fabric BRDF sensitivity. The central estimate uses the nine-fabric mean in the nominal relative-azimuth case, and the conservative range includes all tested fabrics and relative azimuth offsets.

Sensor	Mean Delta [pp]	95% Interval [pp]	Conservative Range [pp]
RGB	−0.35	−0.83 to −0.03	−5.47 to 0.78
Monochrome	−0.68	−1.48 to −0.15	−6.81 to 0.29

### Appendix A.3. SNR Requirement Uncertainty

The SNR-to-detection mapping is bounded by shifting the required SNR curve by −3 dB to +3 dB. This range is used as a conservative perturbation of the required-SNR curve and corresponds to multiplying the linear SNR requirement by 0.71 and 1.41. Figure A8 shows the effect on the WLTP-weighted coverage of all 16 light distributions. The 0 dB case reproduces the baseline coverage values.

**Table A4:** Mean WLTP coverage under SNR requirement shifts. The table reports the mean across the 16 light distributions and the difference relative to the 0 dB baseline.

Sensor	SNR Requirement Shift [dB]	WLTP Mean [%]	Difference to 0 dB [pp]
RGB	−3	44.569	6.292
RGB	−2	42.604	4.327
RGB	−1	40.511	2.234
RGB	0	38.277	0.000
RGB	1	35.896	−2.381
RGB	2	33.404	−4.873
RGB	3	30.810	−7.467
Monochrome	−3	61.389	5.376
Monochrome	−2	59.745	3.731
Monochrome	−1	57.958	1.944
Monochrome	0	56.014	0.000
Monochrome	1	53.907	−2.107
Monochrome	2	51.631	−4.383
Monochrome	3	49.167	−6.847

### Appendix A.4. Reaction-Time Sensitivity Details

Figure A9 shows the full-RoI reaction-time sensitivity with a tight vertical scale. The small full-RoI change confirms that the 1.0 s baseline does not dominate the final WLTP-weighted score.

The three highest RoI risk levels are also evaluated separately. This subset is recomputed for each value of $t_{\mathrm{react}}$, so it is not a fixed subset of the full RoI. The spatial composition changes with the reaction-time parameter, which explains why the high-risk trend can differ from the full-RoI average. Figure A10 shows the high-risk region, Figure A11 reports the corresponding coverage trend, and Table A5 gives the numeric summary.

**Table A5:** High-risk reaction-time sensitivity of the WLTP-weighted coverage score. The table reports the subset formed by the three largest RoI risk levels.

Sensor	$t_{\mathrm{react}}$ [s]	WLTP Mean [%]	Difference to 1.0 s [pp]
RGB	0.5	77.444	−6.776
RGB	1.0	84.221	0.000
RGB	2.0	51.558	−32.663
Monochrome	0.5	83.563	−5.088
Monochrome	1.0	88.650	0.000
Monochrome	2.0	58.993	−29.657

### Appendix A.5. Non-Reconstructive Light-Distribution Characterization

The proprietary IES files are represented through scalar descriptors that cannot reconstruct the original beam patterns. Figure A12 shows the anonymized 1 lx and 3 lx contours for all 16 systems. The underlying road grid extends to 800 m and the plotted range extends to 650 m, so the high-beam 1 lx contours are not clipped.

Figure A13 and Figure A14 summarize the road-plane descriptors. Table A6 reports the corresponding descriptor ranges.

**Table A6:** Non-reconstructive descriptor ranges for the light-distribution dataset. The table reports minimum, median, and maximum values across the 16 anonymized systems.

Beam	Descriptor	Min	Median	Max
LB	Area ≥ 1 lx [m^2^]	2459.75	5167.00	10,560.75
LB	Area ≥ 3 lx [m^2^]	1171.25	2193.75	4071.75
LB	Reach ≥ 3 lx [m]	84.60	120.85	167.60
LB	Width ≥ 3 lx [m]	18.00	32.25	42.00
LB	Horizontal FOV ≥ 3 lx [^∘^]	77.39	95.98	107.95
LB	Angular peak [cd]	49,238.50	71,575.50	87,806.00
LB	90% horizontal width [^∘^]	33.75	46.13	56.50
LB	90% vertical width [^∘^]	6.00	7.25	12.75
HB	Area ≥ 1 lx [m^2^]	7595.25	17,765.38	30,921.25
HB	Area ≥ 3 lx [m^2^]	2465.50	6129.25	10,476.50
HB	Reach ≥ 3 lx [m]	162.60	260.10	347.10
HB	Width ≥ 3 lx [m]	18.00	35.75	49.50
HB	Horizontal FOV ≥ 3 lx [^∘^]	57.87	94.84	106.37
HB	Angular peak [cd]	85,671.75	214,376.63	384,640.00
HB	90% horizontal width [^∘^]	25.50	37.88	45.50
HB	90% vertical width [^∘^]	5.00	9.13	13.25

**Table A7:** Per-system iso-lux width and longitudinal reach. Width is the maximum lateral span of the road-plane region above the threshold, and *L* is the maximum longitudinal reach of the same region.

LIDC	Beam	W 1 lx [m]	L 1 lx [m]	W 3 lx [m]	L 3 lx [m]	W 5 lx [m]	L 5 lx [m]
LIDC 1	LB	40.0	286.1	29.0	163.6	24.0	124.1
LIDC 1	HB	57.0	477.1	32.5	274.1	25.0	212.1
LIDC 2	LB	69.5	244.6	42.0	150.1	32.5	119.6
LIDC 2	HB	41.5	385.1	18.0	217.1	11.0	164.1
LIDC 3	LB	25.5	157.6	18.0	101.1	15.0	79.6
LIDC 3	HB	41.5	322.6	23.5	184.6	17.0	142.1
LIDC 4	LB	42.5	126.1	31.0	87.1	27.0	73.6
LIDC 4	HB	62.5	483.1	37.0	277.6	30.0	214.6
LIDC 5	LB	42.5	264.1	33.0	151.1	29.0	115.1
LIDC 5	HB	55.0	605.6	32.0	347.1	28.0	267.1
LIDC 6	LB	42.5	292.1	33.0	167.6	29.0	128.6
LIDC 6	HB	68.0	601.6	39.5	345.6	30.5	266.1
LIDC 7	LB	36.0	176.6	26.5	113.1	22.5	93.1
LIDC 7	HB	40.5	474.1	26.0	273.1	22.0	210.6
LIDC 8	LB	40.5	240.1	31.5	143.1	27.5	112.1
LIDC 8	HB	53.5	486.6	36.0	282.1	30.5	219.1
LIDC 9	LB	57.5	186.6	38.5	116.6	31.5	94.1
LIDC 9	HB	65.5	319.6	40.5	172.1	32.5	129.6
LIDC 10	LB	43.0	120.1	31.0	84.6	27.0	72.1
LIDC 10	HB	53.5	578.1	35.5	330.6	30.0	253.6
LIDC 11	LB	57.5	186.6	38.5	116.6	31.5	94.1
LIDC 11	HB	76.0	373.1	48.5	217.6	39.0	169.6
LIDC 12	LB	37.5	163.1	27.0	109.6	23.5	91.1
LIDC 12	HB	50.0	382.1	32.0	219.1	26.5	167.6
LIDC 13	LB	68.5	202.6	40.5	125.1	31.5	99.6
LIDC 13	HB	72.0	358.1	43.0	205.1	33.5	158.1
LIDC 14	LB	37.0	158.1	25.0	94.6	22.0	75.6
LIDC 14	HB	47.0	282.6	27.0	162.6	21.5	125.1
LIDC 15	LB	56.0	209.1	37.5	128.1	30.5	101.6
LIDC 15	HB	65.5	433.1	40.0	247.1	31.5	190.1
LIDC 16	LB	56.0	204.6	36.5	128.1	30.5	102.6
LIDC 16	HB	81.5	505.1	49.5	290.1	39.0	223.6

**Table A8:** Per-system horizontal field of view from iso-lux contours. FOV is computed from the road-plane mask above the iso-lux threshold using the lateral angle range of the illuminated region.

LIDC	Beam	FOV 1 lx [^∘^]	FOV 3 lx [^∘^]	FOV 5 lx [^∘^]
LIDC 1	LB	106.5	103.7	102.1
LIDC 1	HB	108.7	103.7	102.1
LIDC 2	LB	101.7	98.4	96.5
LIDC 2	HB	77.2	57.9	54.3
LIDC 3	LB	101.2	86.7	84.6
LIDC 3	HB	95.4	88.7	84.2
LIDC 4	LB	103.3	95.8	90.6
LIDC 4	HB	103.3	97.0	90.6
LIDC 5	LB	101.8	96.2	90.6
LIDC 5	HB	104.5	99.4	94.2
LIDC 6	LB	101.8	96.2	90.6
LIDC 6	HB	101.8	96.2	94.2
LIDC 7	LB	103.7	96.6	90.7
LIDC 7	HB	103.7	96.6	90.3
LIDC 8	LB	99.4	94.4	90.5
LIDC 8	HB	100.5	93.5	89.5
LIDC 9	LB	133.4	108.0	85.8
LIDC 9	HB	99.4	90.2	85.4
LIDC 10	LB	109.0	98.3	95.6
LIDC 10	HB	109.0	98.3	95.6
LIDC 11	LB	98.1	89.9	85.8
LIDC 11	HB	92.1	81.8	79.0
LIDC 12	LB	100.5	94.0	87.0
LIDC 12	HB	102.1	97.6	91.7
LIDC 13	LB	119.8	106.4	101.7
LIDC 13	HB	119.8	106.4	101.7
LIDC 14	LB	86.4	77.4	70.7
LIDC 14	HB	85.8	75.8	68.7
LIDC 15	LB	92.7	81.5	78.9
LIDC 15	HB	93.1	82.8	82.4
LIDC 16	LB	93.4	82.0	78.9
LIDC 16	HB	93.4	82.0	78.9
